# Negotiating gender and creative work in Shanghai’s music industry: female creative workers’ career choices and development

**DOI:** 10.3389/fsoc.2026.1848381

**Published:** 2026-05-28

**Authors:** Yixuan Yang

**Affiliations:** Shanghai Conservatory of Music, Shanghai, China

**Keywords:** career, creative labour, cultural and creative industries, gender equality, music industry

## Abstract

This article examines the career choices and professional development of female creative workers in Shanghai’s music industry and addresses the underexplored gendered dynamics of creative labour in China. Material realities of cultural production have received significant attention concerning artists’ insufficient remuneration and atypical employment; however, how women cope with the complexities of precarious creative work, the pressures of urban life, and gender inequality in China has not been fully documented. This study adopts a qualitative research approach, drawing on 30 in-depth semi-structured interviews and applying thematic analysis. The research findings reveal that career choices are primarily driven by intrinsic motivation, particularly passion for music, while educational background, social networks, economic considerations, and industry prestige also influence decisions. Regarding professional experiences, the urban environment of Shanghai, the creative industry ecosystem, personal experiences, and support resources collectively shape job satisfaction and career development potential. Gender effects are observed through implicit workplace discrimination and conflicts between professional identity and family responsibilities. Career planning is influenced by factors such as marriage and childbearing, salary, growth potential, job stability, working hours, and stress, with family, social networks. These findings suggest that the precariousness of female creative labour in China cannot be understood solely through universal accounts of creative work insecurity. Rather, it is shaped by the interaction between global creative labour inequalities and China’s specific gender norms, labour structures, and policy environment. The study therefore contributes to broader debates on gendered precarity by highlighting how local institutional contexts reshape the experience of creative labour across the life course.

## Introduction

In 2017, the Shanghai municipal government launched an ambitious campaign to transform the city into the “Asian Performing Arts Capital”. Following late-20th-century economic reforms, Shanghai emerged as a frontrunner in creative industry development (Gu, 2014), driven by a strategy that blends state-led initiatives with market-oriented growth (He and Wang, 2019; Ren and Sun, 2012). Shanghai’s unique socio-economic history and political framework provide it with a distinctive “creative edge” compared to other Chinese cities (Liu, 2009). Within the central government’s broader vision, cultural and creative industries serve as the core engine for China’s strategic shift from “Made in China” to “Created in China,” aiming to bolster international competitiveness and cultural soft power (O’Connor and Gu, 2014). In sectors such as music, the state employs a “top-down” investment model, prioritising cultural production as a tool for political communication and propaganda over mere economic gain (Morrow and Li, 2016). Consequently, creative workers within state-owned enterprises face the dual pressure of balancing “social benefits” (ideological alignment) with “economic benefits” (Lin, 2019), often navigating their creative autonomy within strict bureaucratic constraints (White and Xu, 2012).

Against this backdrop of interplay between state power and capital logic, creative workers today increasingly encounter precarious conditions, such as insecure, low wages and excessive workloads. A growing body of research has identified these conditions as a defining feature of contemporary creative labour globally (Banks, 2017; Gill and Pratt, 2008). In this sense, Shanghai is no exception but a part of a broader global dynamic. However, existing scholarship has often framed creative labour precarity through a dichotomy between the Global North and Global South (Lorey, 2015; Munck, 2013), which is increasingly questioned by research calling for more “ex-centric” and globally situated perspectives (Alacovska and Gill, 2019; de Kloet et al., 2020). Within this debate, China occupies an ambiguous position. Rather than fitting neatly into either category, creative workers experience a hybrid form of precarity shaped by both rapid marketisation and strong state governance (Lin, 2019; Chow, 2019), where flexible labour conditions coexist with institutional and political regulation. In this sense, China complicates binary understandings of global creative labour regimes.

Although the municipal government remains focused on attracting creative professionals (Zheng, 2011), the rapid expansion of the cultural economy often rests upon the systemic exploitation of creative labour. While many workers view this field as a site of self-fulfillment (Duffy, 2015), such self-realisation is based on both self-exploitation (Hesmondhalgh, 2010) and broader structural inequalities. These challenges are particularly acute for women, who must navigate persistent gender-based barriers, including the “glass ceiling” (Treviño et al., 2017) and motherhood penalty (O’Brien and Arnold, 2024). Since the 2016 implementation of China’s two-child policy (and the subsequent three-child policy), women have encountered intensified social and economic pressure regarding childbirth, which have further undermined the stability of female employment (Shen and Jiang, 2020). Given this evolving landscape, the career trajectories and agency of female creative workers in Shanghai warrant further examination.

Before proceeding, the concept and scope of “creative workers” needs to be clarified. The term is commonly used to refer to individuals engaged in creative occupations within the creative industries. It is closely related to the notion of “creative class” (Florida, 2003) However, while the “creative class” presents an idealised view of creativity as the engine of urban vitality and economic growth, the term “creative workers” adopts a more critical perspective, foregrounding the instability and exploitative labour relations that characterise much creative work, including low or unpaid wages, short-term contracts, and limited union protection (Chow, 2017). Under the creative intensity approach, the UK government’s creative industries strategy identifies a range of creative occupations (Department of Culture, Media and Sports [DCMS], 2016). A key limitation of this approach is its focus on “front-stage” professionals such as artists, musicians, and dancers, while neglecting many essential “back stage” roles, including museum staff and curators in the visual arts, as well as stage managers and arts administrators in music. By drawing on the Australian Bureau of Statistics’ (ABS) definition of “cultural occupation,” Throsby incorporates backstage creative roles to offer a broader conception of creative labour (Throsby, 2008). This study adopts the creative trident model and focuses on the *specialist workers*, those employed in core creative occupations within the creative industries, including artists and creative professionals (Higgs et al., 2008). Accordingly, “creative workers” are defined here as professional creative individuals engaged in creative labour in the cultural and creative industries. While they are often attributed an idealised role in driving economic growth and urban vitality, they simultaneously experience the precarity and exploitation inherent in creative labour (Chow, 2017).

This paper aims to examine the career choices and professional development of female creative workers in Shanghai’s music industry. It foregrounds women’s values and narratives to identify gender-specific characteristics of creative labour. Accordingly, the study addresses three research questions:

1. What motivates women to pursue careers in the music industry?
2. How do gendered experiences shape their professional lives?
3. How do these workers envisage and plan their future careers?

In this study, 30 female creative workers in Shanghai were selected to explore their experiences and perceptions of both professional and personal lives, providing a basis for theorising how gender intersects and interacts with creative work. Existing scholarship on creative careers has largely examined the gendered dimensions of careers at discrete stages, including career choice (Dubois and Lepaux, 2018), career experience (DiMaggio, 1987), career advancement (Herron et al., 1998), and career transition (O’Neill, 2017; Cannizzo et al., 2025a; Dent, 2019; Been, 2025). It is important to note that the majority of participants are under 30 and in their early career stages, positioning them as a transitional generation navigating the uncertain boundary between youth and adulthood (Bessant, 2020). They are situated within competing contemporary youth narratives identified by Bessant (2020), namely, the “precariat” subject, characterised as insecure and vulnerable, and the “entrepreneurial” young person, who is expected to be self-reliant, adaptable, and responsible for managing their own risks. Concurrently, they can also be understood as a risk-bearing group within conditions of labour precarity in DIY cultural fields (Threadgold, 2018), who may “actively choose poverty”, rejecting conventional middle-class trajectories and redefining success in non-material terms. This study contributes to the literature by tracing a coherent analytical sequence of “career decision-making, career experience, and future career planning”, grounded in an empirical case study of Shanghai’s music industry. Methodologically, this study adopts a qualitative research design based on semi-structured interviews and thematic analysis. Through a thematic analysis of the interview data, the study identifies 11 interrelated themes that illuminate the complex intersections between gender, creative labour, and Shanghai’s urban context. The findings indicate that career choices are primarily driven by intrinsic motivations, particularly participants’ passion for music, alongside factors such as educational path dependence, social networks, economic rationality, and the perceived prestige of the industry. Women’s career experiences are shaped by the combined effects of Shanghai’s urban characteristics, the local creative industry ecosystem, individual experience and resources, and gender. Attitudes towards future career planning vary considerably, reflecting diverse development pathways influenced by factors including marriage and childbearing. In the discussion and conclusion section, relevant literature is subsequently revisited to discuss the specificities of women’s creative labour in China, presenting a clear picture of how they navigate discrimination, precarity, and occupational risks while struggling to unleash their creativity to the fullest.

## Theoretical background: a gendered perspective in China

While gender inequalities have been widely examined, the relationship between women and work still requires contextualised analysis shaped by specific political, legal, and socio-historical conditions. This necessity is reflected in the trajectory of Chinese feminist studies, which has emerged as a diverse field intrinsically connected to the nation’s shifting social and political tides (Barlow, 2004). Recent scholarship within this tradition, such as the frameworks of “patchy patriarchy” (Evans, 2021), the “post-patriarchal era” (Shen, 2011), “neo-patrilocality” (Eklund, 2018), and “coordinated patriarchy” (Liu, 2021) illuminate the intersections of socialist, Confucian, and neoliberal logics. These accounts demonstrate how family-centered social reproduction continues to drive gender inequality in both domestic and professional spheres. Besides, confucian family structures today operate less through explicit doctrinal appeals and more through material practices (housing transfers, occupational sorting, intergenerational care arrangements) and state-backed moral discourses, producing a neo-familialist order in which educated urban women are expected to harmonise professional ambition with intensified filial piety and maternal obligations (Xie and Huang, 2024).

To understand the contemporary tensions within this terrain, it is necessary to revisit the multifaceted forces that have shaped gender equality in China, specifically the interplay between persistent Confucian family structures, the structural transition from a collectivist to a market-driven economy, and the evolving trajectories of state reproductive policies. China’s achievements in women’s labour market participation, widely attributed to the state’s strong commitment to gender equality (Gustafsson and Li, 2000; Nie et al., 2002). Following the establishment of the People’s Republic of China in 1949, Mao Zedong articulated the influential political slogan, “The times are different now. Men and women are the same. Whatever male comrades can do, female comrades can do too” (Huang, 2018). It laid the foundation for challenging gender stratification, promoting gender equality, and safeguarding women’s rights, while making a departure from the patriarchal Confucian social order that had long legitimised male dominance (Wielink, 2019). Despite the persistence of a “patchy patriarchy” (Evans, 2021), the Maoist period radically empowered women by integrating them into the labour market and alleviating domestic burdens through state-sponsored collective services (canteens, childcare, and housing). However, these gains have gradually eroded in the wake of economic reforms. With the abolition of the “*danwe*i” system and socialist policies supporting working women, the retrenchment of welfare and childcare pushed care back into households, exacerbating the tension between work and family and negatively impacting the career development of Chinese women (Cook and Dong, 2011; Ji et al., 2017) with mothers as default providers for childcare (Wallace, 2019). These changes was reflected in declining female labour force participation, widening gender wage gaps, and the persistent underrepresentation of women in enterprise leadership and managerial positions (He and Wu, 2017; Wang and Klugman, 2020; Wei, 2011).

As the market sector expanded, a previously modest motherhood penalty intensified into a significant and widening disadvantage, reflected in career interruptions, reduced promotion opportunities, and wage penalties, whereas fatherhood continues to confer a wage premium. Importantly, wage declines for prospective mothers begin even before childbirth, revealing mechanisms of anticipatory discrimination (Xu, 2023; Guan et al., 2025). These wage gaps are further shaped by structural inequalities across China’s public, private, and collective sectors, which reflect explicit forms of discrimination (Li et al., 2024; He and Wu, 2021). Moreover, these measurable disparities are compounded by the intersection of gender with age, *hukou* status, and education, exposing a systemic pattern of labour misallocation (Guo et al., 2025). While these quantitative gaps provide a visible map of inequality, they are often underpinned by more subtle, implicit forms of discrimination. Women encounter obstacles in hiring, advancement, the maternity penalty, sexual harassment, biased workplace culture, and objectifying language (Liu, 2025). The gradual relaxation of family planning regulations in 2016 has coincided with intensified discrimination against women in the labour market (Cooke, 2017; Leng and Kang, 2022), highlighting the persistence of entrenched normative expectations that women prioritise family responsibilities. Despite high levels of female education and labour force participation, these expectations continue to constrain women’s career trajectories (Connelly et al., 2018). Even for middle-class women aspiring to upward mobility, job stability and perceived work-life balance remain a gendered pathway to social advancement, often through marriage into wealthy families (Xie and Huang, 2024).

Shanghai presents a compelling paradox within China’s gender landscape: while structural inequalities persist, the city remains a pioneer of regional gender equity rooted in its Republican-era cosmopolitanism (Schmid, 2014; Da, 2004). This legacy is evidenced by a distinct tradition of familial egalitarianism (Wai-ming, 2013) and the strategic agency exercised by women in navigating contemporary social constraints and the stigmatisation of highly educated “leftover women” (Ji, 2015). Given this unique synthesis of persistent challenges and progressive norms, Shanghai serves as a critical and innovative site for this study. Consequently, by grounding the gendered perspective in Shanghai’s pineering status and China’s specific historical trajectory, social structure, and contemporary policy environment, this paper seeks to examine the mechanisms through which women’s labour conditions and gender inequalities are reproduced.

## Contextualising creative labour in China: institutional, industrial and spatial dimensions

In contrast to the UK DCMS model (2016), China adopts a more state-led and policy-driven approach, although this is gradually shifting towards a hybrid governance model in which state and market actors increasingly interact in cultural policy under conditions of rapid economic development (White and Xu, 2012). In official Chinese discourse, the term “cultural industries” (*wenhua chanye*) is commonly used, referring to “those industries producing cultural goods on an industrial scale” and conveying the recognition of their growing economic potential (O’Connor and Gu, 2014). The cultural industries are generally understood to encompass three main components: cultural products, cultural services, and intellectual property, which has been progressively institutionalised through national development strategies such as the Cultural Development Plan and successive Five-Year Plans (China Law Translate, 2022). They are defined as “the collection of production activities that provide cultural products and cultural-related products to the public” (National Bureau of Statistics of China, 2018). And the music industry examined in this study falls within both the “core cultural sector” and the “cultural-related sector” as defined in China’s official classification system (National Bureau of Statistics of China, 2018). Within this framework, the state emphasises economic and social benefits while acting as both a promoter and a regulator (Lin, 2019). In addition, China’s state ideology has been compelled to redefine its traditional role and reassert its legitimacy in cultural industry regulation amid rapid economic development, rising cultural identity concerns, the protection of local and ethnic minority arts, and tensions between high and commercial culture (Tong and Hung, 2015). As a result, the opportunities, risks, and career trajectories of creative workers, including musicians, are deeply embedded in this political-legal configuration.

Again this backdrop, the pathways through which individuals enter creative occupations in China are deeply shaped by institutional mechanisms that link education to labour market outcomes. Under the socialist planned economy from the 1950s to the late 1980s, graduate employment was governed by the state through the Job Assignment Policy, whereby individuals were allocated positions according to national priorities rather than personal choice (Bian, 1994). This system established a strong structural connection between education and employment, positioning the state as the primary allocator of labour. Following market-oriented reforms in the late 1990s, the formal job assignment system was gradually abolished, and individuals were increasingly required to compete in an open labour market (Wang, 2020). However, the structural allocation of labour is not really eliminated. In contemporary China, the linkage between education and occupation continues to be mediated through highly competitive examination systems, most notably the *Gaokao*^1^ and, for arts students, the *Yikao*.^2^ The *Gaokao* is widely described as the “single wooden-pole bridge” to higher education and urban life (Cheng and Hamid, 2025; Liu and Helwig, 2020), with examination scores largely determining institutional tier and field of study, and consequently shaping employment prospects and life chances (Lu, 2023). *Yikao*, alongside the *Gaokao*, determine which majors and career pathways are realistically attainable, operating as a parallel threshold that filters access to creative disciplines (Lin and Weatherly, 2024). In this case, educational choices are often made strategically to maximise admission prospects or future income rather than to pursue artistic aspirations (Li, 2025), reinforcing a system in which creative labour is channelled through institutionalised pathways. Therefore, while the overt mechanisms of state job allocation have receded, the structuring of career opportunities persists through the education system, shaping both access to and stratification within China’s creative industries.

As the empirical focus of this study, Shanghai represents one of China’s most advanced and strategically positioned cultural and creative hubs. As an early and proactive adopter of creative-industry policies, the city established itself as a leading centre for cultural production and performing arts ahead of central government mandates (O’Connor and Gu, 2014; Wei and Jian, 2009). Supported by sustained state investment and policy initiatives, Shanghai hosts a dense concentration of theatres, music venues, production companies, and cultural institutions, making it a critical site for examining the organisation of creative labour. However, while Shanghai exhibits a distinctive “creative edge” not easily replicated elsewhere (Liu, 2009), its cultural economy remains shaped by a hybrid governance model in which market mechanisms coexist with state-led investment and regulatory oversight. This rapid institutional ascent has also engendered significant structural risks, including acute career instability and intensifying cost pressures that jeopardise both new enterprises and the retention of creative talent (Zhong, 2011). In the music sector in particular, state-owned enterprises in music industry remain embedded in a “top-down” investment structure that frames cultural production as an instrument of political governance rather than solely a site of market-driven accumulation (Morrow and Li, 2016). At the level of labour, these institutional arrangements translate into heightened precarity. The survival pressures faced by the creative workers in Shanghai, including low wages and job precarity, echo the structural vulnerabilities observed in Western creative labour markets (Hesmondhalgh and Baker, 2010). These challenges are compounded by a specific intersection of high-cost urban living (Chow, 2017) and the discourse of creativity, creating “an increasingly flexible, informal, and precarious condition” (Lin, 2021). This combination of strong institutional support and heightened labour insecurity makes Shanghai a particularly revealing case for understanding how structural conditions shape creative careers.

Looking at the contemporary Chinese music industry specifically, it is often described as undergoing a transition from a state-led system to a market-oriented one, alongside the emergence of a digital, platformised, and state-conditioned labour regime, in which creative workers have gained new access to audiences, while simultaneously becoming more dependent on highly concentrated platforms, short-video ecosystems, copyright governance, and censorship (Montgomery, 2009; Zhang and Negus, 2024; Qu et al., 2021). Compared to many Western markets, independent and self-releasing musicians have become increasingly significant within the Chinese music industry, but this apparent “democratisation” has largely been absorbed into platform logics (Qu et al., 2021). That said, platformisation and digitalisation are not the primary focus of this article. As most of the subjects examined here work in the field of live music performance (with only one participant employed in a music technology company producing music for streaming and video games), greater attention must be paid to the market structure of live performance and its practitioners. Live music performance remains fragile and highly spatially contingent, shaped by factors such as venue closures, urban politics, political pressures, and post-COVID disruptions (Gu et al., 2021; Zhang and Xiao, 2023; Fung and Zhang, 2023). Existing research on the Chinese live music market tends to focus on niche genres, particularly independent music and rock, as well as non-mainstream venues such as live houses. In contrast, relatively little attention has been paid to mainstream music genres, classical music, and state-owned performances in concert halls and theatres. By examining both state-owned and non-state-owned live performances across a broader and more diverse range of contexts, this study seeks to address this gap.

Within this industrial context, research on music labour in China that directly engages with gender remains limited. As discussed above, recent studies have made behind-the-scenes music workers, such as planners, operators, and venue entrepreneurs in China, more visible. However, these studies rarely adopt a gender perspective. Despite the growing body of research on gender and creative labour in worldwide, there remains a notable lack of scholarship, in Chinese or focusing on China, on the career choices and development of female creative workers. One notable example shows that female jazz singers in Shanghai face institutionalised inequalities, manifested in male-dominated training pathways and exclusion within elite professional networks (Jiang, 2024). For women, these conditions are further intensified by gendered expectations related to appearance, age, and family roles (Ji, 2015; Xie and Huang, 2024; Xie, 2019), as well as unequal access to professional networks and opportunities (Zhang et al., 2021). Despite this, gender remains underexplored in existing studies of China’s music labour. This article addresses this gap by examining how the precariousness of creative labour intersects with broader gender inequalities within China’s creative economy for female creative workers. And the following literature review section provides an empirically grounded analysis of how gender, precarity, and creative work intersect within the global dynamic.

## Literature review

### The special nature of creative labour

The distinctive character of creative industries gives rise to unique forms of creative labour, which provides an important foundation for this study. Scholars have identified several key features of creative labour, including aspirational labour, free labour, immaterial labour, and mobility, which continue to be explored in empirical research. Aspirational labour (Duffy, 2015), is “a highly gendered, forward-looking and entrepreneurial enactment of creativity,” closely associated with the maxim “DWYL” (Do What You Like) and orienting workers toward future career prospects. Over the past 40 years, aspirations among young women have intensified (McRobbie, 2011). Mendes (2022) further describes digital feminist labour as precarious, immaterial, aspirational, and affective. Similar to Higgins (1989) Self-Discrepancy Theory, the distinction between “actual, ideal and ought selves” elucidates the psychological mechanisms underlying identification and aspirational labour. Hesmondhalgh (2010) highlights the precarious conditions of creative workers, noting that many tolerate low wages and long hours in pursuit of self-realisation, a phenomenon described as “*self-exploitation.*” Hesmondhalgh and Baker (2010) similarly analyse free labour in television, recording, and magazine industries, identifying common expressions of victimisation, anxiety, and isolation. Immaterial labour, as defined by Lazzarato (1996), refers to “labour that produces the informational and cultural content of the commodity.” Research on mobility in creative labour spans three dimensions: social, spatial, and occupational. Brook et al. (2022) examine social mobility and “openness” in creative occupations, highlighting a mobility crisis. Spatial mobility studies have focused on Chinese creative cities such as Beijing, Shanghai, and Hong Kong. Chow (2017) explores the mobility of Hong Kong creative workers in Shanghai and Beijing, while Tse (2022) considers job insecurity, suggesting that mobility can serve as a power to resist insecurity. Career mobility, as examined by Chow (2019), can be understood across macro (inter-city migration), meso (intra-city relocation), and micro (daily movement) levels. These characteristics of creative labour often coexist in practice, intersecting to shape the dynamics of the creative industries and the experiences of creative workers.

The unique characteristics of creative labour are frequently examined within a broader social context. Oakley et al. (2017) analyse spatial inequalities in cultural employment resulting from the “*London effect,*” which McCall (2018) observes that economic inequality within this sector is widely accepted as a normative condition. Focusing on recruitment practices, Brook et al. (2020) examine how gender, ethnicity, educational achievement, and geography intersect with class-based inequalitiesFrom an economic and labour market perspective, artists face challenges, including precarious gig work, high levels of self-employment, and reliance on temporary contracts (Woronkowicz, 2025). Woronkowicz et al. (2020) note that non-profit arts organisations frequently rely on flexible labour rather than salaried employment. Self-employment is also more prevalent among creative workers than in most other occupations (Feder and Woronkowicz, 2023). Moreover, location within a metropolitan art market exerts a significant influence on artistic freelancers (Woronkowicz and Noonan, 2019). The role of cities in shaping creative labour conditions is further supported by Brook et al. (2025), who show that sustaining a livelihood solely through creative work is more difficult for individuals based outside London. In addition, Hancock and Tyler (2025) demonstrate that recognitive instability, combined with affective and socio-economic precarity, creates challenging working conditions for freelance and self-employed artists.

### Gender studies of creative workers

Gender research on creative workers in the arts and cultural industries can be broadly organised into four areas: diversity, intersectionality, gender equality, and motherhood. Studies on diversity examine the demographic composition of cultural organisations (Topaz et al., 2019; Cuyler, 2015), racial and ethnic inequalities among arts practitioners (Stein, 2019), and the relationship between workforce diversity, leadership, and compensation (Cuyler, 2017). Regarding intersectionality, research analyses how gender intersects with age, race, and class to shape opportunities and inequalities within the cultural sector (LeBlanc and Sheppard, 2022; Tatli and Özbilgin, 2012; Talwar, 2010). Extensive scholarship reveals deeply embedded gendered structures in the cultural industries, from the standards of evaluation in art history (Nochlin, 2017) to the gender gaps in arts institutions (Treviño et al., 2017). Structural barriers are reinforced through stereotypes, microaggressions, and “glass ceilings” (Burke, 2024; Herron et al., 1998), while dominant models of masculine entrepreneurship and leadership continue to marginalise women and constrain their career development (Tams, 2003; Carey, 2013; Carr and Van Raalte, 2025; Nixon and Crewe, 2004). These inequalities are particularly pronounced in the music industry, where macho workplace cultures persist and women often undertake additional emotional and affective labour (Scharff, 2022; Gross, 2022; Barna, 2022). Research shows that women’s gender identities act as barriers in certain genres, including rock music and electric music (Bayton, 1989, 1997; Coates, 1997; Bourdage, 2010; Parsley and Johansson, 2025). Motherhood constitutes another key focus in gender studies of creative labour. Research highlights the structural incompatibility between creative work and parenting, which is often individualised and managed by women themselves (O’Brien and Arnold, 2024; O’Brien, 2025). These challenges vary considerably across national contexts (Arnold et al., 2025).

### Career choices and experiences in the music industry

Regarding the career choices and professional experiences of female creative workers, empirical research has examined how gender shapes both career entry and experiences (Dubois and Lepaux, 2018; DiMaggio, 1987). Research on career trajectories highlights how women’s life-course transitions and material circumstances influence mid-career decisions (O’Neill, 2017). The accumulation of job stress, precarity, and limited institutional support often creates critical pressure points that lead to exit from the industry (Bienvenu, 2004). Been (2025) demonstrates that career trajectories reflect structural inequalities, with women earning less than men across their careers, and immigrant, female, and young workers exhibiting the highest exit rates. Similarly, the careers of women artists tend to become increasingly precarious with age (Coulangeon et al., 2005). Recent studies also address career interruptions and re-entry. Cannizzo et al. (2025b) examine the challenges faced by women and gender non-conforming music workers in career breaks, showing how they navigate gendered barriers within the industry. Dent (2017) further shows that women’s devalued economic positions make it difficult to reconcile creative work with motherhood, exposing a persistent tension between the creative sector’s egalitarian ideals and the lived realities of its workforce (Dent, 2017).

Beyond these structural and gendered dynamics, recent scholarship points to a broader transformation by using age as a new analytical dimension under late capitalism. Threadgold (2018) shows that young people engaged in DIY scenes must continuously negotiate the normalised nexus of employment, unemployment, and underemployment while struggling to sustain creative autonomy and maintain space for artistic practice. In such contexts, success is defined through the capacity to continue creative engagement instead of income or the traditional perception of “career” (Threadgold, 2018), while Arnold and O’Brien (2025) find that younger generations increasingly prioritise mental health and work-life balance alongside creativity. Rizkidarajat et al. (2025a) propose the concept of “double precarity,” where young people simultaneously confront unstable labour markets and the pressures of maintaining authenticity and legitimacy within DIY cultural fields. Rizkidarajat et al. (2025b) further show that attempts to build DIY music careers are often undermined by incomplete creative infrastructures, forcing young musicians to adopt adaptive but ultimately unsustainable strategies within an “unfinished” creative city. In addition, as Sutopo et al. (2021) note, the active resistance to market commodification and neoliberal creative economy policies—by sustaining values of autonomy and authenticity among young musicians—is heterogeneous. These studies suggest that career choices and development in the music industry for young people are shaped by both structural constraints and shifting work motives, in which economic survival, creative commitment, mental health, and ethical orientations are deeply intertwined. In conclusion, this study contributes to research on creative labour by addressing the lack of contextually grounded analyses of female creative workers’ careers in China’s music industry.

## Materials and methods

Data were collected between 2024 and 2025 over an eight-month period through semi-structured, one-to-one interviews. While initial access to participants was facilitated through professional contacts and personal networks, the study employed a purposive sampling design to ensure that recruitment aligned with the core research questions.

A total of 30 participants were selected based on three eligibility criateria: (1) self-identification as a woman; (2) at least 1 year of work experience in Shanghai; and (3) self-identification as a “creative worker.” Beyond these baseline requirements, I actively monitored for diversity across analytically relevant categories to avoid the risks of homophily and utilized specific sub-criteria to address the study’s three research questions.

- Organisational and Employment Diversity (Q1 and Q2): I included participants from various types of arts organisations and employment statuses (full-time, part-time, and intership) to capture diverse entry pathways and labour conditions.
- Occupational Roles (Q2): Professional Roles were deliberately varied (e.g., performer, administrator, technical worker) to examine gendered segmentation and differentiated opportunities for advancement.
- Life-Stage and Relationaship Status (Q2 and Q3): I ensured a mix of participants across different ages, relationship statuses, and parental statuses, enabling me to track how professional identities intersect with shifting personal lives.
- Migration Background (Q1- Q3): I contrasted the narratives of Shanghai natives with non-native residents to explore how migration background and the city’s high living costs influence their creative career.

Each interview lasted over 1 h and was conducted in Chinese, audio-recorded, transcribed, and subsequently translated into English. All interviews were anonymised, with each participant assigned a numerical identifier (see Table 1). I allowed discussions to develop organically, using open-ended questions, such as “Is there anything distinctive about the creative work environment in Shanghai?”, to encouraged participants to share nuanced perspectives.

**Table 1:** Basic profile of the 30 interviewees.

Interviewee	Age range	Types of arts organisations	Position	Employment status	Relationship status	Parity	Shanghai native
No. 1	20–25	Musical Theatre Production Company	Assistant Stage Manager/Director	Part-time/Internship	Single	0	Yes
No. 2	26–30	Performance Company/Music Therapy Organisation/Music Training Institution	Performer/Music Therapist/Teacher	Full-time	In a relationship	0	No
No. 3	20–25	Performance Company/Arts Venue/Music Training Institution	Performer/Arts Manager/Teacher	Part-time/Internship	In a relationship	0	No
No. 4	26–30	Musical Theatre Production Company/Music Tech Company	Assistant Stage Manager/Sound Engineer	On probation	Single	0	No
No. 5	20–25	Music Licensing Company	Social Media Operations Specialist	Full-time	Single	0	Yes
No. 6	20–25	Troupe/Performance Company	Performer	Full-time	In a relationship	0	No
No. 7	20–25	Performance Company	Stage Designer	On probation	Single	0	Yes
No. 8	26–30	Musical Theatre Production Company	Assistant Stage Manager/Sound Engineer	Part-time/Internship	Single	0	No
No. 9	26–30	Musical Theatre Production Company/Arts Venue	Stage Designer	Full-time	In a relationship	0	Yes
No. 10	65–70	Troupe/Arts Venue	Stage Manager /Performer	Full-time	Married	1	No
No. 11	20–25	Musical Theatre Production Company/Arts Venue	Art Manager	On probation	Single	0	No
No. 12	26–30	Troupe/Arts Venue	Composer/Sound Engineer	Full-time	In a relationship	0	No
No. 13	20–25	Performance Company	Arts Manager/Assistant Stage Manager	On probation	Single	0	Yes
No. 14	20–25	Record Label/Music Production Company/Music Tech Company	Assistant Stage Manager/Props Master/Operations Specialist	Part-time/Internship	Single	0	No
No. 15	20–25	Performance Company/Musical Theatre Production Company	Assistant Director/Social Media Operations Specialist/Arts Manager	Part-time/Internship	Single	0	No
No. 16	20–25	Musical Theatre Production Company/Music Tech company	Assistant Stage Manager/Operations Specialist	Part-time/Internship	Single	0	No
No. 17	26–30	Performance Company/Arts Venue/Symphony Orchestra	Arts Manager/Performer	Part-time/Internship	Single	0	No
No. 18	26–30	Musical Theatre Production Company/ Music Tech Company	Producer/ Stage Manager	Full-time	Single	0	Yes
No. 19	31–35	Performance Company/Music Training Institution	Performer/Teacher	Full-time	Single	0	No
No. 20	26–30	Arts Venue	Arts Manager	Full-time	Single	0	Yes
No. 21	55–60	Arts Venue	Arts Manager	Full-time	Married	1	Yes
No. 22	26–30	Arts Venue	Arts Manager	Full-time	In a relationship	0	Yes
No. 23	26–30	Arts Venue	Arts Manager	Full-time	Single	0	Yes
No. 24	36–40	Musical Theatre Production Company	Producer/Stage Manager	Full-time	Single	0	No
No. 25	26–30	Symphony Orchestra	Arts Manager	Full-time	Single	0	No
No. 26	26–30	Arts Venue	Arts Manager	Full-time	In a relationship	0	No
No. 27	36–40	Performance Company	CEO	Full-time	Married	2	No
No. 28	40–45	Theatre	Arts Manager	Full-time	Married	1	No
No. 29	40–45	Theatre/Symphony Orchestra	Arts Manager	Full-time	Married	1	Yes
No. 30	51–55	Troupe/Music Training Institution	Conductor/Teacher	Full-time	Married	1	No

While a detailed overview of participants is provided in the appendix, several key characteristics of the sample are worth highlighting here. The majority of interviewees are under the age of 30, with over two-thirds falling within the 20–30 age range, indicating that the findings primarily reflect early-career experiences within the first decade following formal education.

In terms of employment roles, a significant proportion of participants occupy organisational positions, such as arts managers, producers, and administrative staff within cultural institutions. Although the sample also includes performers, composers, and conductors, these roles are often combined with or embedded within organisational structures rather than representing fully independent freelance careers.

The sample further spans a diverse range of genres and organisational settings within the music and performing arts industries. These include musical theatre production companies, performance troupes, symphony orchestras, arts venues, music technology firms, record labels, and music training institutions. In terms of genre, participants are primarily engaged in musical theatre, Western classical music (e.g., orchestras), and popular music-related sectors, with relatively limited representation of other forms such as traditional Chinese music.

These structural features of the sample provide important context for interpreting the findings, as experiences are likely to vary across career stages, organisational environments, and genre-specific industry practices. As such, the findings should be understood as particularly reflective of early-career and institutionally embedded creative workers within specific segments of the music and performing arts sector.

For data analysis, I adopted a thematic analysis approach following Braun and Clarke (2006). This bottom- up method allowed codes and themes to emerge directly from the data rather than being guided by the researcher’s theoretical assumptions (Braun and Clarke, 2006). Six analysis stages involved:

1. Familiarisation with the data: Transcripts of the audio recordings were read repeatedly to become familiar with and organise the data.
2. Generating initial codes: Relevant segments of text were identified and assigned concise codes, which were continuously reviewed and refined.
3. Identifying themes: Codes were examined and grouped into potential themes relevant to the research questions, with attention to similarities, overlaps, and relationships between themes.
4. Reviewing themes: The coherence and relevance of codes and themes were evaluated, leading to the refinement, addition, or removal of themes where necessary.
5. Defining and naming themes: Each theme was clearly defined in terms of its distinctive features.
6. Producing the report: The final themes were organised into a coherent analytical framework and presented in the reporting of the research findings (Braun and Clarke, 2006).

This rigorous process generated 1,225 reference points and 11 overarching themes: 5 themes addressed Q1, 4 themes addressed Q2, and 2 themes addressed Q3. Finally, I must carify four methodological considerations. First, given the individualised nature of gendered experiences, qualitative methods were deemed more appropriate than quantitative approaches, and the sample was not intended to be statistically representative. Second, I followed the principle of theoretical saturation (Morse, 1995), continuing recruitment until all variations and “negative case” perspectives were fully represented within or between the purposive participant categories mentioned above. Third, measures were taken to ensure validity and reliability through systematicity and consistency (Gibson and O’Connor, 2003). Fourth, the study adhered to strict ethical standards. I recognise that these interviews collected sensitive data, including discussions on discrimination and family conflict, and I maintained rigorous protocols for informed consent, privacy protection, and data confidentiality.

## Research findings

**Figure 1:**
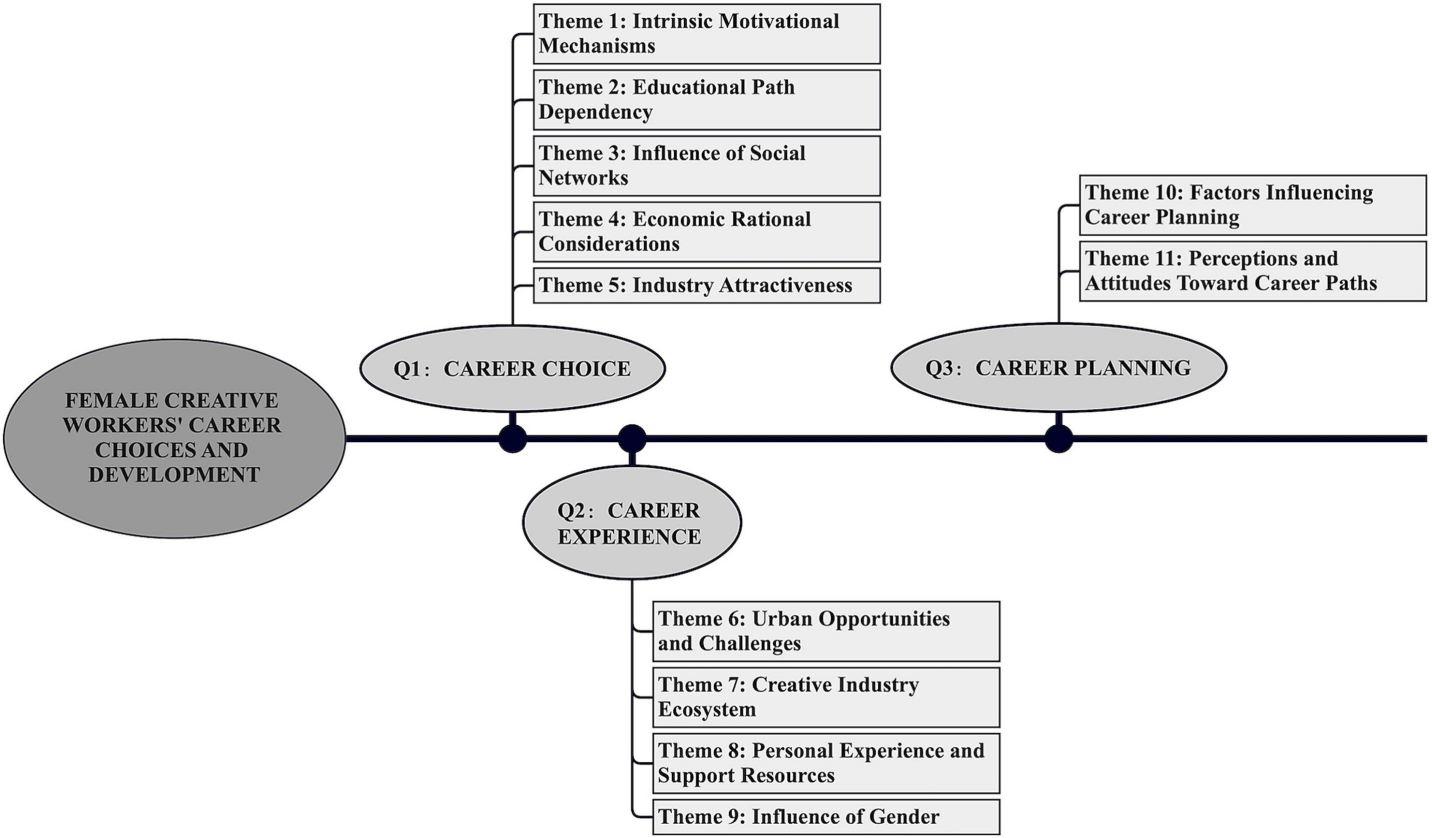
Concept map illustrating the 11 themes derived from the interviews.

This section presents the findings derived from the thematic analysis. Eleven key themes were identified and organised around the three research questions (see Figure 1). These questions are structured along a “past-present-future” timeline, providing a coherent framework for understanding their career development.

### Motivations to pursue careers in the music industry

When asked what motivates them to pursue a career in the music industry, participants responded with one or more of five themes: *Intrinsic Motivational Mechanisms*, *Educational Path Dependency*, *Influence of Social Networks*, *Economic Rational Considerations*, and *Industry Attractiveness*.

*Intrinsic Motivational Mechanisms* reflect the extent to which women’s choices to enter the music industry are driven by personal interests and values. This theme comprises five sub-themes: *Interest and Passion*, *Talent and Skill Development*, *Influence of Idol Culture*, *Curiosity-Driven Motivation*, and *Achievement-Driven Motivation*. 70% of participants identified interest and passion as their primary motivation, often tracing back to childhood experiences such as listening to pop music, learning instruments, participating in extracurricular classes, or joining choirs. Career choices were deeply intertwined with artistic education, regardless of whether participants had formal training or were self-taught enthusiasts.

*Influence of idol culture* is particularly pronounced in musical theatre. For example, Participants no. 8 described becoming an assistant stage manager after watching a musical theatre reality show, noting that entering the industry allowed her to “be a fangirl.” She suggested that admiration for musical theatre actors is a common motivation among young women in this field. Curiosity and the pursuit of accomplishment also shaped career decisions, while *Talent and Skill Development* reflected participants’ confidence in the alignment between their abilities and their work, as well as their desire to refine specific skills. As Participants no. 12 stated:

When I'm making music, the thoughts, melodies, and lyrics that come out of my mind are as natural as breathing to me, so I'm very confident in myself. (Participants no. 12, composer and sound engineer)

*Educational Path Dependency* refers to the extent to which female creative workers’ career choices are shaped by their formal education. This theme comprises four sub-themes: *College Major Assignment After the Gaokao, Job Assignment Policy, Educational Sunk Costs,* and *School Resource Referrals*.

Within the *Gaokao* system, where examinaton score largely determine access to specific majors, early academic allocation can have long-term occupational consequences. Firstly, the mismatch between personal interests and academic specialisation reported by the participants reflects a distinct career trajectory driven primarily by the high-stakes examination system. The reason behind this is that students often select subjects and majors strategically to maximise admission prospects or expected income rather than to pursue genuine interests (Li, 2025). In addition, this phenomenon also reflects a broader pattern of structural and geographical inequality in China, where diverging artistic trajectories among Shanghai and non-Shanghai respondents are observed. For many participants from outside Shanghai, the *Gaokao* was the defining moment that led them to the city and ultimately sparked their journey into the music industry.

I had no idea of the music industry before. I went to a concert for the first time after entering university through the *gaokao* with an arts administration major. So, I chose this job because I graduated with a music degree, not because I was passionate about music.” (Participants no. 29, arts manager)

As examined by Lin and Weatherly (2024), majors are structured by region, class, gender, and family resources, rather than by individual preference alone. Meanwhile, art candidates from remote areas are admitted at substantially lower rates than their urban counterparts (Zhang, 2025).

*Job assignment policy* illustrates the distinctive features of career choices for creative workers in China during a specific historical period. Participants no. 14, born in the 1950s, recalled being assigned to work at an opera house after graduation, reflecting the state’s authority over individual career paths. Other participants highlighted the influence of educational sunk costs, namely the investment of time, money, and experience, on their career decisions. *School Resource Referrals* also played a role, with some participants entering the music industry through industry–university collaboration projects and internship programmes. The credibility and recommendations provided by universities offered tangible advantages to young women, who often lacked social capital and competitiveness when entering the music industry.

*Influence of social networks* highlights the role of personal relationships, including family, friends, teachers, and other close contacts, in shaping career choices. Many participants described key opportunities as serendipitous, such as a symphony orchestra hiring unexpectedly, a chance encounter with an industry professional, or a sudden performance opportunity, all of which were mediated by social connections. Family expectations and support also played a significant role, providing emotional, financial, and networking assistance that influenced career decisions. For example, Participants no. 22 described navigated family opposition and bore economic pressures alone to demonstrate the viability of her career in the music industry.

*Economic rational considerations* refer to how living costs and financial security influence career choices, particularly in Shanghai’s high-living-cost environment. Approximately one-third of participants reported prioritising salary and job stability, with some emphasising the necessity of retaining any available position. Uneven distribution of employment opportunities within the music industry often prompted shifts from “front-stage” roles to “backstage” positions, such as from performer to music administration. Career interruptions caused by the termination of arts projects or closure of institutions further compelled practitioners to seek alternative employment. Participants no. 29 described being forced into a career transition following the conclusion of an eight-year tour.

*Industry attractiveness* represents a key motivation for female creative workers at the early stages of their careers. The appeal of the music industry often stems from public admiration for musicians and artists, as well as a romanticised view of the work involved, reflecting participants’ responses to the ethos of “Do What You Like” (Duffy, 2015) and supporting the view that “the need for beauty and prestige is balanced against the risks of leaving full-time work to the more precarious labour market for poetry, art, and theatre” (Brook et al., 2020). Yet, in a market as oversaturated and hyper-competitive as Shanghai’s, realising creative ideals is far from a guaranteed outcome for all. According to several participants, discrepancies between idealised expectations and workplace realities, particularly repetitive or routine tasks, can undermine the sense of contribution and passion and contribute to decisions to leave the industry, which aligns with Astin’s (1984) argument.

### Gendered experiences of precarious creative lives

In response to the second research question, “How do gendered experiences shape your professional lives?”, four themes were identified: *Urban Opportunities and Challenges*, *Creative Industry Ecosystem*, *Personal Experience and Support Resources*, and *Influence of Gender*.

*Urban Opportunities and Challenges.* Shanghai, as one of China’s most vibrant and mature music performance markets, offers unique career opportunities for female creative workers, often in comparison with cities such as Beijing, Nanjing, Guangzhou, and Qingdao (Participants no. 12, 13, 17). Participants no. 17 noted that the city’s highly developed market operations make creative work more professional and efficient. The artistic atmosphere and cultural literacy of Shanghai also play a subtle but important role, with Participants no. 19 observing that the arts are integrated into daily life. In this tolerant, open, and culturally diverse environment, the high concentration of creative communities and talent affords participants a robust cultural network, enhancing their professional identity and sense of belonging (Florida, 2003). However, women face structural challenges. For non-locals in Shanghai, the high cost of rent is a major pressure, with Participants no. 3 explaining that financial strain forces some professionals to resign, contributing to high staff turnover in arts organisations. The financial burden also limits the ability of creative workers to pursue hobbies outside of music, necessitating personal sacrifices (Participants no. 12). The study further found that, across all career stages, Shanghai natives are more likely to remain in the music industry, whereas workers from other regions are more prone to exit.

*Creative Industry Ecosystem* exerts a profound influence on women’s professional lives, with some experiences common across the broader sector rather than unique to the music industry. First, wages are a prominent concern. Several participants reported that salaries in the music industry are insufficient to sustain a living (Participants no. 1, 3, 4). Free labour is also common, particularly in the early stages of careers (Participants no. 1, 7), and many supplement their income through part-time or secondary jobs, often relying on music teaching to support their stage ambitions (Participants no. 16). This finding echoes with the widely recognised “hidden costs” of creative work and economic inequalities (McCall, 2018). Second, the work schedule diverges from the typical 9-to-5 model, as most performances take place in the evenings or on weekends, often requiring additional social engagements after work (Participants no. 4, 8, 24, 25). Third, employment instability and project-based work are recurring challenges (No. 1, No. 3, No. 16, No. 23). For project-based and freelance workers, the supposed autonomy of the music industry translates into an absence of institutional support. While a sense of precarity is also pervasive among those in traditional employment, evidenced by high turnover rates in state-owned music halls (Participants no. 26), the nature of this insecurity differs. For those within the state system, precarity manifests more as a high-pressure working environment rather than financial risk, as they still benefit from stable, state-backed salaries and institutional financial protections.

Fourth, heavy workloads and intense work pressure are widely reported in the industry. (Participants no. 8, 15, 26) Even interns are expected to remain on call, while performance periods often require continuous work from morning to night with little rest (Participants no. 4). Pressure rarely derives from formal evaluation systems but instead from unpredictable interpersonal dynamics and unforeseen incidents during live performances (Participants no. 19, 23). As several interviewees noted, anxiety stemming from the uncertainty of stage work is a defining feature of the profession (Participants no. 6, 14). Fifth, relational labour further intensifies these pressures. Constant interpersonal engagement increases emotional and psychological strain, particularly for introverted workers, and often reduces job satisfaction (Participants no. 2, 4, 8). Sixth, emotional labour is perceived as unavoidable, especially for roles such as producers and stage managers who act as mediators between performers and teams (Participants no. 3, 8, 9, 17). This labour is highly gendered, as women are expected to demonstrate superior empathy and communication skills, leading to cumulative fatigue and emotional exhaustion (Participants no. 3, 5, 17). Seventh, professional rivalries are framed as structural consequences of resource scarcity and status hierarchies rather than personal hostility and jealousy (Participants no. 8, 15, 19). Respondents highlighted the extreme concentration of income among a top-tier minority against a precarious majority, alongside deep-seated resentment stemming from status differentials between front-stage and back-stage workers (Participants no. 1, 4, 5, 13). This finding aligns with Everts et al.’s (2022) observation that, while new acts must strategically accumulate milestones to build reputation, they face a “winner-takes-all” landscape where success is exceptionally rare.

Top musicians have the vast majority of the audience, resources, and performance opportunities. The rest of us, ordinary people, can only compete with each other in the remaining 10% of the market. That's the harsh reality of the music industry. (Participants no. 3, performer, arts manager, and teacher)

Furthermore, the research findings indicate that intense competition in Shanghai’s music industry exacerbates practitioners’ anxiety and psychological stress, echoing previous work linking the cognitive instability of live performers to economic vulnerability (Hancock and Tyler, 2025).

*Personal experience and support resources* examines job satisfaction, affective orientations towards work, and the role of family support in managing work–life boundaries. Firstly, more than half of the participants reported that job satisfaction was primarily derived from their intrinsic love of music. As Participants no. 1 noted, “If I could do this (artistic creation) full-time, then going to work would be a very happy thing for me.” This attachment to music strongly shaped participants’ sense of professional value and self-identity, particularly through live performance. However, as Lin (2019) observes, the autonomy of creative workers within Chinese state-owned cultural enterprises is tightly constrained by the party-state’s ideological priorities and the imperative of “being creative for the state”. Consequently, job satisfaction derived from artistic contribution is accompanied by constant negotiations between individual creative autonomy and the rigidities of the bureaucratic structure (White and Xu, 2012).

Secondly, a sense of dedication was frequently emphasised by participants. For many, dedication functioned both as a motivation to endure long-term precarity and as a source of self-satisfaction that enhanced psychological well-being (Participants no. 10). However, younger participants were more critical of this discourse. Participants no. 4 described appeals to dedication as a form of psychological manipulation that legitimises low-paid labour. The frequent use of phrases such as “it’s worth persisting for what you love,” “you have to be mentally prepared to do this,” and “you need a spirit of dedication” in participants’ accounts demonstrates the pervasiveness of aspirational and affective labour (Mendes, 2022), as well as self-exploitation (Hesmondhalgh, 2010).

Thirdly, family support emerged as another key factor shaping job satisfaction and career continuity, especially for women with children. Within the allocation of household and parenting responsibilities, men often do not share equally in domestic tasks or child-rearing (Hooks, 2015), which influence women’s status within both the family and the public sphere (Maharaj, 1995). Adequate support from partners or parents facilitated career persistence and post-childbirth returns, while its absence often intensified pressure, contributing to career stagnation or exit. Zhou (2020) demonstrates that maternal employment among young urban Chinese women has become moralised, such that being a “good mother” entails not only wage work but also intensive caregiving, a norm rooted in the socialist legacy of full employment and shaped by the biographical experiences of their mothers. In the low-paid music sector, childcare costs frequently exceed individual earnings, forcing women to weigh continued employment against withdrawal from paid work (Participants no. 29). Participants nos. 14 and 27 highlighted the decisive role of familial support in enabling their return to work after childbirth. Participants no. 27 described career interruptions due to childbirth, referring to it as “a sweet burden.” It also confirms the presence of the motherhood penalty and challenges in returning to work after childbirth within Shanghai’s music industry (Cannizzo et al., 2025b; Dent, 2017; Coulangeon et al., 2005).

*Influence of gender* examines gendered professional experiences shaped by social roles, cultural expectations, and personal values. Gender inequality in the music industry is primarily manifested through occupational segregation, the glass ceiling, and the commodification of women’s appearance. First, Over 60% of participants perceived pronounced occupational gender segregation in Shanghai’s music industry. Women are frequently channelled into supportive or administrative roles within arts organisations, while technical positions such as lighting and sound remain male-dominated (Participants no. 17, 21, 31). Gender segmentation is also evident across music genres, with rock and independent music fields described as predominantly male (Participants no. 10, 11). Secondly, the glass ceiling was widely identified, with leadership and decision-making positions largely occupied by men, while women remain concentrated in lower- or mid-level roles with limited access to institutional power (Participants no. 3, 10, 24). Intersectional dynamics further compound inequality, as younger women are often perceived as lacking authority or managerial competence, undermining their professional legitimacy (Participants no. 8). Thirdly, participants also highlighted the commodification of female musicians’ appearance. In some performance contexts, women are treated as visual assets, with hiring criteria explicitly prioritising attractiveness over musical competence. Participants no. 4 noted that certain engagements specify requirements such as being “sexy” or “beautiful,” while performances at hotels or private events may mandate gendered dress codes, including cheongsams or short skirts. As she reflected:

Some performance opportunities only want female musicians to attract attention. As long as you look pretty and can play, that is enough. They don't care what you play or your musical skills. If you want to make money, you must accept it. (Participants no. 4, performer, arts manager, and teacher)

Lastly, some participants perceived a gradual improvement in gender equality, particularly in Shanghai, which was described as relatively open and tolerant compared with other Chinese cities (Participants no. 5, 9, 28). According to Participants no. 24, professional women in Shanghai experience comparatively less pressure regarding age, marriage, and childbirth than in other cities.

### Towards an (un)sustainable creative career future

In response to the question “How do you plan your future careers?”, two themes emerged: *Factors Influencing Career Planning*, and *Perceptions and Attitudes Toward Future Career Paths*.

*Factors influencing career Planning* could be understood across six dimensions. First, childbearing-related constraints were widely perceived as potential threats to career continuity. Many participants sought to preserve professional autonomy by postponing, rejecting, or minimising marriage and childbearing responsibilities (Participants no. 6, 8, 14). This career and fertility dilemmas are primarily driven by two factors. On the one hand, gender-based parental leave policies, coupled with discriminatory hiring practices, have led women to believe that having multiple children and achieving career success are simply incompatible (Zhou, 2018). On the other hand, according to Liu (2021), it reflects a structural dilemma between career advancement and personal aspirations, shaped both by socially constructed work-family boundaries and by the conflicting values attached to private and professional identities.

Second, assessments of current employment, including salary, career advancement opportunities, and job stability, collectively played a decisive role in shaping future plans. Thirdly, family support exerted a dual influence. Material and emotional support enabled long-term engagement in creative work, whereas its absence often precipitated forced career adjustments or exits (Participants no. 16). Family responsibilities also generated pressure and moral obligation. Participants no. 18 noted that a strong sense of responsibility towards her family might compel her to return to her hometown for future employment. Women’s identification as “daughters” often entails greater emotional and caregiving expectations, a burden intensified by the legacy of the one-child policy (Participants no. 8, 24).

Fourth, cities played a decisive role in shaping future career planning. Shanghai was widely perceived as offering a more developed music market and greater employment opportunities, while creative workers in smaller cities faced heightened economic and professional precarity (Participants no. 4, 18). Besides, urban middle-class families increasingly expect daughters to adapt their careers and residential locations to maintain parental support while remaining compatible with the social and spatial requirements of their future in-laws (Eklund, 2018).

Fifth, several participants emphasised the importance of personality-job fit. Misalignment between personal traits and occupational demands undermined perceptions of career sustainability and prompted considerations of exit or career change (Participants no. 15, 24). Finally, future career planning was also influenced by perceptions of labour market change. As Participants no. 5 reflected:

With the development of AI, more creative or repetitive labour will be replaced, and some people will lose their jobs. In the future, we may need to learn how to use tools or rethink the direction of our work. (Participants no. 5, social media operations specialist).

The above findings indicate that the career development and personal lives of female creative workers are closely intertwined, which applies to women across different life stages, aligning with O’Neill (2017) and Bienvenu (2004) regarding drivers of career exit and transition, with a background of patriarchy that persists in fragmented and rearticulated forms (Evans, 2021).

*Perceptions and attitudes toward future career paths*. Participants expressed diverse orientations towards their future careers, reflecting differentiated values and coping strategies. Four dominant attitudes emerged: confusion, realism, active exploration, and persistence in music. Some participants reported uncertainty and a lack of long-term career direction, emphasising the unpredictability of creative careers (Participants no. 8, 19). The reported frustration at their limited control over career trajectories is prevalent globally (Brook et al., 2025; Banks, 2017; Taylor, 2018; Dubois and Lepaux, 2018). Others adopted a pragmatic stance, viewing work primarily as a means of economic survival (Participants no. 13, 21). Consistent with Arnold and O’Brien (2025), the younger generation of creative workers tends to prioritise work-life balance over purely artistic ideals. A smaller group demonstrated an exploratory orientation, seeking to experiment with different roles to broaden future possibilities (Participants no. 7, 9), reflecting proactive resistance strategies within a precarious creative economy (Woronkowicz, 2025; Brook et al., 2025). By contrast, many participants remained committed to the music industry, valuing creativity and emotional fulfilment over stability (Participants no. 18, 30). These attitudes corresponded to five anticipated career trajectories. First, some participants considered exiting the music industry due to low pay and instability (Participants no. 5, 6). Second, others viewed creative work as a long-term lifestyle, aiming to sustain artistic practice and cultural influence rather than pursue conventional career advancement (Participants no. 14, 30). Third, returning to education emerged as a strategy for overcoming career stagnation, with some participants planning postgraduate study or overseas training (Participants no. 12). Fourth, several participants articulated open-ended planning, remaining receptive to multiple future pathways (Participants no. 3). Finally, non-standard employment arrangements, including freelancing, self-employment, and portfolio careers, were particularly attractive to younger workers, who valued flexibility, autonomy, and control over their time and creative practice (Participants no. 8).

## Unpacking the precariousness of gendered creative labour in China

Gendered experiences of creative labour in the Chinese music industry resonate with structural inequalities seen globally in the creative industries while also reflecting distinct local socio-cultural influences.

Aligning to a broader global trend, gender inequality among creative workers in Shanghai’s music industry manifests in several ways: gender discrimination (Treviño et al., 2017; Herron et al., 1998; Nochlin, 2017), gender segregation within arts institutions and across musical genres (Bayton, 1989; Bayton, 1997; Coates, 1997; Bourdage, 2010; Parsley and Johansson, 2025), and macho culture practices (Scharff, 2022; Nixon and Crewe, 2004; Carr and Van Raalte, 2025). Firstly, participants reported that women were predominantly employed in planning or marketing roles, which are culturally coded as feminine and emphasise communication, organisation, and coordination skills. Men, in contrast, were mostly responsible for stage-technical roles, defined by operational precision, mechanical skill, and analytical judgment, reflecting culturally coded masculine abilities. Regarding music genres, rock and independent music, associated with rebelliousness and masculinity, are male-dominated, while women are steered towards more mainstream or “softer” genres in this study. Secondly, according to participants’ observations, while women can attain middle and senior management roles, men overwhelmingly occupy final decision-making positions (Burke, 2024; Herron et al., 1998), consistent with findings in the global arts and cultural sector. This pattern was noted across conservatories, leading symphony orchestras, musical theatre companies, and performance organisations. Additionally, the intersection of gender and age, where young women are often perceived as lacking sufficient managerial experience in theatres (Participants no. 8). Thirdly, female creative workers are expected to undertake, or are perceived as more suited to, emotional and relational labour, consistent with Barna (2022), who found that women bear substantial unrecognised emotional labour in maintaining workplace relationships and resolving conflicts. Lastly, female musicians are frequently required to adhere to specific appearance or dress codes to secure performance opportunities, reflecting a “process of sexualising women as objects of heterosexual desire” and “standardising feminine appeal” (Connell, 2013, p. 113). Generally speaking, some participants were acutely aware of these unequal structures (Gross, 2022), while others justified them in the name of the “fairness” promoted by the idealised creative industries (Tams, 2003).

Echoing with research on subtle and structural forms of gender discrimination in China’s labour market, this study highlights the implicit exclusion of women in the music sector, with the project-based, network-dependent nature of the music labour market further obscuring potential pay gaps. Consistent with Zhang et al. (2021), recruitment processes in Shanghai’s music industry appear to exhibit subtle biases, with women facing lower probabilities of securing interviews despite comparable qualifications. Furthermore, the study supports the argument of Bai et al. (2023) concerning the persistence of an “unexplained gender gap,” as women are frequently marginalised from core technical training and high-level decision-making positions. This exclusionary pattern is further reinforced by pro-natalist policies and socio-cultural expectations surrounding women’s domestic roles. Following the implementation of the two- and three-child policies, employers’ expectations regarding women’s domestic responsibilities have intensified, contributing to what He et al. (2022) describe as a subtle “potential fertility” penalty in hiring and task allocation. This dynamic is reflected in the present findings: even young single female creative workers report experiencing discrimination and career-related stress linked to anticipated marriage and childbirth (Participants no. 25, 29), while others experienced guilt or psychological burden arising from physical factors such as menstrual cycles (Participants no. 28). In Shanghai, where maternity-related benefits may impose additional costs on employers, institutional arrangements can further reinforce women’s marginalisation in recruitment and career progression (Yin and Wei, 2022). Ultimately, as Xiao and Asadullah (2020) argue, such discrimination is embedded in broader structural gender norms that shape professional hierarchies.

This precariousness becomes particularly pronounced when examined through the lens of age and life-course transitions (Threadgold, 2018; Rizkidarajat et al., 2025a). A significant majority of the participants are young female creative workers grappling with a lifecycle-specific instability, where maintaining a long-term career in music becomes an escalating challenge. And participants who had children consistently highlighted the indispensability of intergenerational support in sustaining their low-paid creative careers (Chen et al., 2011). This reflects broader Chinese family norms, where informal kinship networks compensate for limited public childcare provision (Zhong and Peng, 2020). However, access to such support is uneven, shaped by income, family structure, and generational dynamics, meaning that the ability to remain in the music industry is highly stratified. Many participants reported exiting the field due to childbirth and the prohibitive cost of private childcare. These dynamics reveal how the music industry amplifies gendered vulnerability by coupling irregular labour structures with critical life-stage pressures, making exit more likely for women than men.

Building on the global trends of creative precarity and structural inequalities in China’s labour market outlined above, the following section demonstrates how gendered precarity is concretely produced and experienced among female music practitioners in Shanghai. This precarity is simultaneously site-specific, industry-bound, and deeply gendered.

Firstly, although gender was not a direct factor for starting a music career in this study, it operates as an indirect and cumulative force across educational trajectories. The One-Child Policy fundamentally reshaped the trajectories of urban daughters, who, as the sole beneficiaries of parental investment, became the primary vehicles for family upward mobility (Xie, 2019). There exists a persistent presupposition among participants that music, an expensive career path often deemed financially unstable and low-paid, is a luxury reserved for urban middle-class daughters, predicated on the patriarchal assumption that they need not serve as the household’s primary income earner. Yet, as Fong (2004) notes, this “only hope” status produces immense pressure and competitive stress, generating a tension between economic rationality and the perceived attractiveness of the music industry in terms of career choices. Despite the formal gender neutrality of the *Gaokao* and *Yikao* (Cheng and Hamid, 2025; Liu and Helwig, 2020), professional trajectories are still shaped by a gendered logic embedded within music training pathways. For instance, certain genres such as jazz remain informally exclusionary toward women from the outset (Jiang, 2024). As a result, Chinese female creative workers in music do not enter the industry on equal terms but through already filtered and constrained pathways, marking the early-stage production of a gendered vulnerability specific to the music field.

Secondly, this early differentiation is reinforced through gendered expectations surrounding “appropriate” career pathways. Women’s ambitions are frequently redirected toward feminised and socially respectable professions aligned with Confucian-derived norms and middle-class ideals (Xie and Huang, 2024). This dynamic is directly reflected in the interview, where participants reported parental pressure to abandon unstable performance careers in favour of more secure school positions or higher-paying roles in the legal and financial sectors. While these “safe” careers offer better work-life balance, they often come at the cost of long-term professional growth and financial parity (Xie and Huang, 2024) In response, some women actively resist these expectations by delaying or rejecting marriage and childbirth, prioritising career autonomy, echoing Chow (2019) findings on singlehood among female creative workers in Shanghai. However, this resistance is itself structured by social stigma, particularly the discourse of “leftover women” (Ji, 2015). Consequently, female creative workers navigate a profound conflict between their career ambitions and the pragmatic imperative to ensure domestic stability, rooted in traditional gender expectations.

Thirdly, Shanghai presents a paradoxical gender landscape. On the one hand, its relative openness compared to other Chinese cities has fostered progressive gender norms and familial egalitarianism (Schmid, 2014; Da, 2004; Wai-ming, 2013). In this study, Shanghai provided a fragile safety net for women in the music industry. Participants also noted comparatively lower pressures regarding age, marriage, and childbirth, suggesting a more tolerant environment for professional women to pursue their artistic ideals. At the same time, this relative gender equality and the city’s creative atmosphere are mutually reinforcing, underpinned by robust cultural networks (Florida, 2003). On the other hand, the persistence of institutional and spatial inequalities, particularly those linked to *hukou*, housing, and access to urban resources (Guo et al., 2025), tethers individuals to the city despite its high cost of living and intense competition. Many participants with children expressed an inability to leave Shanghai due to the long-term benefits it provides for the next generation’s access to education and welfare. As a result, gender inequality in Shanghai produces a city-specific form of constrained openness.

## Conclusion

Amid the rapid growth of Shanghai’s creative industries and the booming music market, this study explored the career choices and development of female creative workers. Through in-depth interviews with 30 participants and thematic analysis, 11 key themes were identified. This study examines the precariousness of female creative labour in Shanghai’s music industry by situating it within both global patterns of creative labour inequality and the specific institutional context of China. It first demonstrates that gendered inequalities in Shanghai resonate with broader trends in the creative industries, including occupational segregation, gendered divisions of labour, and the persistence of male-dominated decision-making structures. These dynamics are further compounded by subtle and structural forms of discrimination in China’s labour market, as well as by the intensification of “potential fertility” penalties under pro-natalist policy shifts. Finally, it highlights how precarity intensifies across the life course, particularly through motherhood and unequal access to intergenerational support, often leading to women’s exit from the music career. Building on this, the study develops a context-specific analysis of how gendered precarity is produced across different stages of musical careers in Shanghai. It shows that inequalities emerge early through gendered educational pathways, are reinforced by normative expectations around “feminised” careers, and are reconfigured within Shanghai’s paradoxical context of relative openness and structural constraint. Taken together, these findings reveal a form of precarity that is simultaneously global in structure and locally embedded, shaped by the intersection of gender norms, policy legacies, and the unstable nature of creative labour.

However, several limitations should be acknowledged. First, the focus on Shanghai may constrain the generalisability, making it difficult to fully capture the experiences in other Chinese cities. Future research could adopt comparative approaches across different regions. Second, because of the evolving nature of career reflections over time, longitudinal studies could thus provide a more systematic understanding of how perceptions and experiences develop over the course of a career.

## Data Availability

The datasets presented in this article are not readily available due to participant confidentiality and ethical considerations. Requests to access the datasets should be directed to the corresponding author.

## Footnotes

1 The *Gaokao, or* National College Entrance Examination in China, is a highly competitive and high-stakes examination with profound and lasting effects on students’ lives (Cheng and Hamid, 2025).

2 The *Yikao,* also known as the Arts College Entrance Examination (ACEE), operates alongside *Gaokao* cultural scores as a dual threshold for entry into arts universities and creative careers (Lin and Weatherly, 2024).
